# Glucocorticoid action in the anterior pituitary gland: Insights from corticotroph physiology

**DOI:** 10.1016/j.coemr.2022.100358

**Published:** 2022-08

**Authors:** Michael J. Shipston

**Affiliations:** Centre for Discovery Brain Sciences, Edinburgh Medical School:Biomedical Sciences, University of Edinburgh, Edinburgh, EH89XD, UK

**Keywords:** HPA axis, Hypothalamic pituitary adrenal axis, CRH, Corticotrophin releasing hormone, AVP, Arginine vasopressin, CORT, corticosterone

## Abstract

The anterior pituitary is exposed to ultradian, circadian and stress-induced rhythms of circulating glucocorticoid hormones. Glucocorticoids feedback at the level of the pituitary corticotroph to control their own production through multiple mechanisms. This review highlights key insights from analysis of the dynamics of rapid and early glucocorticoid feedback that reveal both non-genomic and genomic mechanisms mediated by glucocorticoid receptors. Importantly, a common target is control of electrical excitability and calcium signalling although non-genomic effects may also involve control of hormone secretion distal to calcium signalling. Understanding the mechanisms and functional consequences of pulsatile glucocorticoid signalling in the anterior pituitary promises to elucidate the role of glucocorticoids in health and disease, as well as identifying potential diagnostic and therapeutic targets.

## Highlights

• Anterior pituitary corticotrophs are exposed to dynamic changes in circulating glucocorticoids.
• Glucocorticoid feedback is via non-genomic and genomic mechanisms over different time domains.
• A common emerging mechanism is through control of cell excitability and calcium signalling.
• Defining the mechanisms and dynamics is key to diagnosing and treating stress-related disorders.

**Figure 1:**
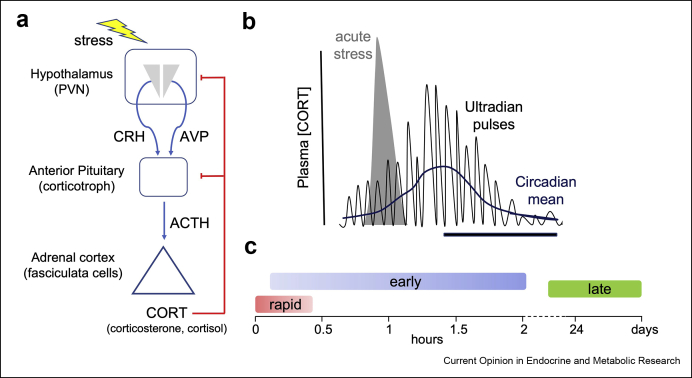
**Dynamics of glucocorticoid output of the HPA axis.a**) Schematic of the key components of the hypothalamic -pituitary-adrenal (HPA) axis. Corticotrophin releasing hormone (CRH) and arginine vasopressin (AVP) are the major hypothalamic neuropeptides that stimulate corticotrophs to release adrenocorticotrophic hormone (ACTH) that stimulates glucocorticoid (CORT: cortisol in man, corticosterone in rodents) from the adrenal gland. CORT feedback at the anterior pituitary and hypothalamic level control output of the HPA axis. **b**) Plasma levels of glucocorticoids display an ultradian rhythm (approximately 1 pulse per hour) and circadian rhythm. In response to acute stress plasma glucocorticoids are elevated with the amplitude and time course dependent on the type and duration of stressor. **c**) Different time domains of glucocorticoid regulation of the anterior pituitary corticotrophs.

Glucocorticoid hormones, the output of the hypothalamic–pituitary–adrenal (HPA) axis (Figure 1a), are potent regulators of physiological function, playing important roles in energy homeostasis and adaptation to stressors [1, 2, 3∗, 4]. In humans and other mammals, plasma glucocorticoid levels (cortisol in man, corticosterone in rodents) display dynamic oscillations that differ in both amplitude and periodicity across multiple time domains [2,3,5]. Under non-stressful conditions, glucocorticoids display hourly (ultradian) pulses whose amplitude can range over at least an order of magnitude varying from tens of nM to several 100 nM with ‘free’ levels in plasma determined by steroid binding proteins. This ultradian rhythm also displays an underlying 24 h (circadian) rhythm, resulting in the highest glucocorticoid levels at the start of the activity phase (Figure 1b). These oscillations are important for the physiological effects of glucocorticoids on target tissues and disruption of these rhythms is associated with cardiovascular, metabolic, endocrine. and neurological disorders [1, 2, 3∗, 4]. Furthermore, in response to a stressor, glucocorticoid levels are further elevated with variable duration and intensity. Importantly, glucocorticoids regulate their own production through negative feedback regulation of the HPA axis acting at the level of both the brain and anterior pituitary gland over different time domains [3,6, 7, 8, 9∗∗].

**Figure 2:**
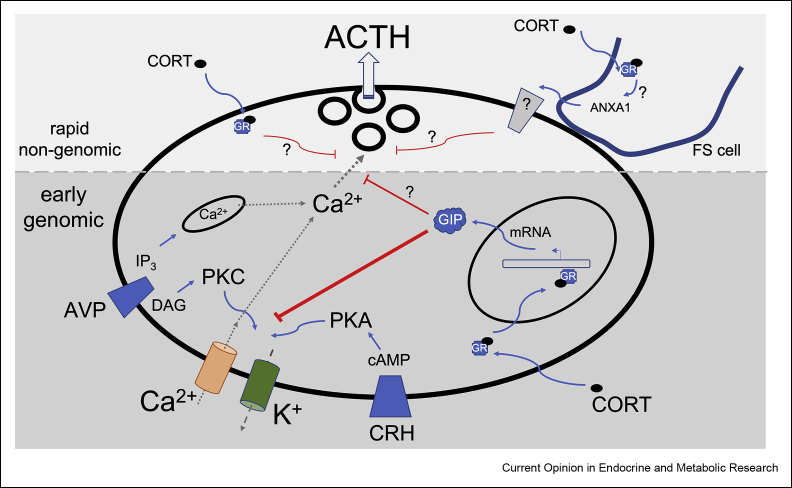
**Mechanisms of rapid and early glucocorticoid feedback inhibition of ACTH secretion from anterior pituitary corticotrophs.** CRH and AVP activate distinct G-protein coupled receptors linked to the cAMP/PKA and DAG/PKC pathways, respectively that control membrane excitability and calcium influx via regulation of a diverse array of ion channels. Early glucocorticoid inhibition involves glucocorticoid receptor (GR) activation of gene transcription resulting in induction of glucocorticoid induced proteins (GIP). These proteins largely act downstream of cAMP/PKA and PKC signalling to control membrane excitability and calcium influx, although additional pathways may be involved. Two non-genomic pathways have been described, including a rapid cell autonomous pathway and a paracrine pathway involving control of annexin1 (ANXA1) release from folliculostellate (FS) cells. Mechanisms underlying rapid inhibition are unknown but have been proposed to be distal to calcium influx.

By using paradigms that more closely parallel the pulsatile behaviour of glucocorticoids, studies are beginning to define mechanisms of glucocorticoid regulation of anterior pituitary corticotroph physiology. This review will focus on mechanisms of glucocorticoid regulation that occur in the early (typically 10 min tõ 2 h) and rapid (< 10 min) time domains following glucocorticoid exposure (Figure 1, Figure 2). In addition, new insights into the potential mechanisms underlying regulation of corticotroph biology following chronic stress and long-term glucocorticoid treatment will be highlighted. As glucocorticoids also control other hypothalamic–pituitary axes, these studies may also provide new insight into glucocorticoid regulation of other anterior pituitary cells, including somatotrophs and gonadotrophs [10,11].

## *Anterior pituitary cells are exposed to dynamic changes in corticosterone over multiple time scales*

The ready accessibility of the anterior pituitary to circulating glucocorticoids is essential for feedback inhibition of the HPA axis, as well as the generation of ultradian rhythms [2,3,7]. Elegant experimental and mathematical modelling in rats revealed that a delay of ∼10 min between feedforward release of ACTH from anterior pituitary corticotrophs and early feedback of glucocorticoid at the corticotroph [12,13] is required for ultradian rhythms. Furthermore, in humans and mammals, the anterior pituitary corticotroph is a major site of feedback in the timescale of minutes to a few hours (rapid and early feedback) [2,3,6,7]. In addition, both the brain and anterior pituitary may be subject to late feedback over a timescale of hours to weeks, for example, during chronic stress or with the widespread use of glucocorticoid therapy.

Remarkably, we in fact know very little about the dynamics and importance of glucocorticoid oscillations at the level of anterior pituitary cells themselves. In addition, while many anterior pituitary cells, including corticotrophs, are also controlled by pulsatile release of hypothalamic neuropeptides, the relative timing of these central and peripheral inputs is likely critical to the output of the system as many *in vivo* studies have shown [3]. This dynamic behaviour has important implications for how we study glucocorticoid action at the anterior pituitary and our ability to distinguish physiological from pharmacological mechanisms. Indeed, controversies in the field regarding both the functional importance of rapid and early glucocorticoid feedback, as well as the underlying cellular mechanisms involved, likely result from a number of different considerations. These include: i) the wide array of *in vitro* models used, ranging from clonal cell lines to dispersed primary cells and pituitary segments; ii) an over reliance on supraphysiological levels and durations of secretagogue iii) pharmacological levels of glucocorticoid at ‘clamped’ levels rather than using pulses that more closely correspond to the situation *in vivo*.

## Early glucocorticoid feedback: Evidence for genomic regulation and control of cellular excitability

### Glucocorticoids inhibit ACTH release evoked by episodic pulsatile secretagogue stimulation via GR-mediated genomic mechanisms

In a number of previous studies in which the effect of early glucocorticoid feedback on ACTH secretion evoked by pulses of secretagogue in perifused isolated anterior pituitary cells, or pituitary segments in an attempt to investigate dynamics *in vivo*, several key features are consistently established [14, 15, 16, 17, 18, 19] (Figure 2). Firstly, endogenous glucocorticoids (corticosterone, cortisol), as well as synthetic glucocorticoids (dexamethasone, RU28362), inhibit ACTH secretion evoked by physiological pulses of the major ACTH secretagogues, CRH and AVP (Figure 1, Figure 2), within 10 min of steroid exposure. Importantly, glucocorticoids have no effect on basal ACTH secretion or ACTH content in this timeframe. Glucocorticoid inhibition is sustained after immediate withdrawal of the CORT, but is reversible. For example, inhibition of episodic stimulated ACTH release remains for ∼ 1 h following a 30 min pulse of 100 nM dexamethasone. However, the rate of reversibility is dependent on the duration, concentration and timing of glucocorticoid and secretagogue pulse. Similar features of glucocorticoid inhibition are also seen for other secretagogues, such as oxytocin [15,18,19]. Importantly, glucocorticoid inhibition is mimicked by GR-specific agonists but not by inactive steroids such as cortisone or other steroids including oestrogen or testosterone, but blocked by GR receptor antagonists such as RU38486. Glucocorticoid inhibition of CRH-evoked ACTH secretion is blocked by inhibitors of transcription (such as actinomycin D or DRB) and translation (puromycin is much more effective than cycloheximide), suggesting glucocorticoid induction of mRNA and protein expression is required for inhibition. Of course, one potential caveat with use of inhibitors of transcription and translation is that a protein that rapidly turns over, but is not induced by glucocorticoid *per se*, is required for early glucocorticoid inhibition. However, washout of the reversible translation inhibitor puromycin *following* a short pulse of glucocorticoid results in a subsequent inhibition of episodic CRH-evoked ACTH release. This supports that glucocorticoid induction of de novo mRNA and protein synthesis is key [15].

Unequivocal evidence that glucocorticoid inhibition of AVP (or oxytocin) induced ACTH release requires de novo mRNA or protein induction is not clear. Indeed, actinomycin D and puromycin alone both inhibited AVP evoked ACTH secretion in response to repeated pulses in perifused pituitary segments, although DEX had no further inhibitory effect [18], suggesting that AVP may preferentially regulate release of newly synthesised ACTH. Intriguingly, potential differences in the mechanism of early glucocorticoid inhibition between CRH- and AVP-evoked ACTH secretion was revealed in such perifusion studies. For example, CRH (or cell permeable cAMP analogues) applied simultaneously with the start of the corticosterone exposure delayed subsequent inhibition of CRH-, but not AVP-evoked ACTH secretion [19]. This suggests that glucocorticoids regulate multiple pathways to control secretagogue-evoked ACTH release. The majority of these studies support a model by which glucocorticoids inhibit ACTH secretion distal to second messenger production, as glucocorticoids have no significant effect on cAMP or IP3 generation and secretion evoked by cAMP agonists or PKC activation are similarly inhibited [15]. These key features of early glucocorticoid inhibition have largely been recapitulated in a number of different systems, using static assays, including the ATt20 D 16:16 mouse corticotroph cell line [20]. Furthermore, similar genomic mechanisms, in this time domain, have been reported in static systems for glucocorticoid inhibition of other pituitary hormones such as prolactin within 15 min [21].

### A role for CORT regulation of cellular excitability and calcium signalling

The first convincing evidence that CORT inhibits calcium signalling came from studies in AtT20 D 16:16 corticotrophs where DEX pretreatment inhibits CRH-induced calcium influx and ACTH secretion [22] in a transcription/translation dependent mechanism. In contrast, ACTH secretion evoked by direct activation of L-type voltage dependent calcium channels with BayK8644 was largely resistant to DEX inhibition; BayK8644 could largely restore CRH-stimulated ACTH secretion in the presence of DEX. Other pharmacological manipulations that result in membrane depolarisation can also counteract DEX inhibition in both AtT20D 16:16 and native corticotrophs [20,22, 23, 24] suggesting that cellular excitability and secretagogue induced calcium influx are key targets for early glucocorticoid feedback. However, it should be noted that in AtT20D16:16 cells, glucocorticoids are also reported to inhibit non L-type voltage-gated calcium currents that do not appear to be functionally coupled to the control of ACTH release, an effect that diminished with cell passage [25].

More recent studies support a role for glucocorticoid-dependent regulation of membrane excitability and calcium signalling under conditions that more closely follow the dynamics of pulsatile secretagogue and glucocorticoid exposure in native corticotrophs. In male murine corticotrophs isolated from pomc-GFP mice, to allow visual identification of corticotrophs in dispersed pituitary cell culture, corticosterone (CORT) exerts complex effects on membrane excitability in both a time and secretagogue dependent fashion [26]. Under basal conditions murine corticotrophs display low frequency (< 1 Hz), spontaneous large amplitude calcium-dependent action potentials with typical duration of ∼50 ms and mean resting potential ∼ - 50 mV [27, 28, 29]. Exposure to 100 nM CORT had no significant effect on spontaneous activity up to 2.5 h after addition of CORT although a biphasic effect on resting membrane potential is observed. CORT had no effect on membrane potential within 5 min of exposure however after 90 min, a significant hyperpolarisation of ∼10 mV was observed that had returned to control by 2.5 h. A 3 min pulse of CRH/AVP, at concentrations that mimic stress induced levels in the portal circulation (200 pM/2 nM), results in a sustained (∼15 min) increase in excitability, associated with both depolarisation of resting membrane potential and an increase in both single spike frequency as well as a transition to pseudo-plateau bursting [27]. The transition to bursting is largely driven by CRH and is dependent upon large conductance calcium- and voltage-activated potassium (BK) channels. Bursting is largely abolished by pharmacological inhibition of BK channels with paxilline or genetic deletion of the pore forming subunit in murine corticotrophs. In contrast, increased spike frequency is largely driven by AVP.

CRH/AVP was able to depolarise the membrane potential to a similar extent in the presence of CORT at all time points, suggesting that mechanisms controlling initial depolarisation are not affected. However, CORT inhibited CRH/AVP induced bursting, but not spiking, within 10 min and suppressed both bursting and spiking at longer time points. Using mathematical modelling and dynamic clamp to reintroduce BK currents in CORT treated cells restored CRH/AVP evoked bursting. This suggests CORT controls CRH-dependent regulation of BK channel function. Such reciprocal regulation of BK channels by cAMP and glucocorticoid signalling has been demonstrated in both corticotrophs and recombinant systems expressing BK channels through competing actions of PKA and protein phosphatases closely coupled to the channels [30,31]. Mice with a global genetic deletion of BK channels display hyporesponsiveness of the HPA axis to acute restraint stress, although both the pituitary and brain are involved [32]. Importantly, the effect of CORT on corticotroph excitability is not solely mediated via regulation of BK channels. Firstly, BK channels do not control corticotroph resting membrane potential [27,28] and secondly, CORT also suppressed CRH- and/or AVP- induced spike activity in corticotrophs in which BK channels were genetically deleted [26]. Taken together these data support a model in which CORT regulates corticotroph excitability over multiple time domains of early glucocorticoid inhibition, and via a variety of different mechanisms. This would provide mechanisms for glucocorticoids to inhibit evoked ACTH release, irrespective of whether secretion is driven primarily via the CRH or AVP pathway that may be important in response to different stressors.

Bursting and spiking are critically important for the control of calcium influx. Bursting in a number of pituitary cell types, including somatotrophs, being a more efficient mechanism to promote secretion, although this can be compensated by high-frequency spiking [33,34]. Indeed, over the same time scale CORT inhibits calcium signalling evoked by repeated pulses of CRH or AVP in corticotrophs [35]. Using isolated rat corticotrophs transduced with lentivirus expressing the calcium reported GCAMP6 allowed the effect of corticosterone on calcium signalling, evoked by multiple episodic pulses of CRH and/or AVP, paralleling prior secretion studies.

Exposure of corticotrophs to 3 min pulses of secretagogue, every 30 min, revealed considerable heterogeneity across the population in terms of the amplitude, kinetics and duration of the evoked calcium response. Importantly, each individual cell showed a stereotypic response to the same stimulus. Similar heterogeneity in the time course of corticosterone inhibition of secretagogue evoked calcium signals was also seen, depending on secretagogue used. For example, in corticotrophs, exposed to repeated 3 min pules of 200 pM CRH, approximately 40% of cells showed a significant reduction (by at least 50%) of the CRH-evoked calcium response 5 min following corticosterone exposure. In this same time domain, less than 15% of cells exposed to either 2 nM AVP or a combined 200 pM/2 nM CRH/AVP stimulus were significantly inhibited by corticosterone. In contrast, following 30 min of corticosterone exposure, more than 70% of cells showed a significant suppression of CRH- or AVP- evoked calcium, whereas only ∼40% of cells exposed to repeated combined CRH/AVP stimulus were calcium signals significantly attenuated after 30 min. Thus, a critical feature is the ‘recruitment’ of a larger proportion of cells that are sensitive to CORT inhibition with time, depending on the stimulus. Whether the lower proportion of AVP-stimulated cells that are initially inhibited by CORT in part reflects the relative balance of calcium release from intracellular vs. influx from extracellular calcium stores remains to be resolved. However, an effect of glucocorticoid downstream of IP3-mediated calcium release would fit with previous data that glucocorticoids have little effects on calcium levels induced by oxytocin [17] or the first phase of secretion in response to supraphysiological AVP stimulation due to intracellular calcium release [36]. Moreover, the relative resistance to CRH/AVP evoked calcium signalling may contribute to AVP induced ‘escape’ of glucocorticoid inhibition [37].

### Time course and nature of glucocorticoid induced proteins

Inhibitors of transcription and translation block many aspects of early glucocorticoid inhibition although this has not been verified or tested in all cases. Although a wide array of glucocorticoid induced proteins have been reported in pituitary cells ranging from calcium binding proteins [38,39], small GTPases [40], protein kinases [41,42] and ion channel subunits [24,43,44], bone-fide glucocorticoid induced proteins essential for early glucocorticoid inhibition remain largely elusive.

Furthermore, it is often commonly ‘assumed’ that glucocorticoid effects faster than ∼30 min are mediated via non-genomic mechanisms [45]. However, in the pituitary, studies that have examined the dependence of transcription and translation reveal that a genomic mode of regulation can occur within ∼10 min after initial exposure to glucocorticoid and GR translocation to the nucleus. Such timing fits with broad estimates for de novo transcription/translation of a ‘typical’ eukaryotic protein encoded by a 10 Kb gene the combination of transcription (100 nt/s), splicing, mRNA export and translation/folding (10 aa/s) that could be achieved within ∼10 min. However, several mechanisms may allow even faster rates comparable with fastest times for glucocorticoid inhibition of evoked secretion that have been determined of ∼5 min. Steroids have been reported to produce fully functional proteins within 5 min [46] in eukaryotic cells. Furthermore, transcription and translation has also been reported in the nuclei of eukaryotic cells that may allow very rapid transcription as seen in prokaryotes (< 1 min, [47]). In addition, increasing evidence supports that a large family of small proteins (< 50 aa) can be rapidly induced, many of which encode proteins that regulate membrane signalling complexes [48]. Clearly, whether such rapid *genomic* mechanisms are important for glucocorticoid regulation in the pituitary warrant further investigation.

## A role for non-genomic glucocorticoid signalling via cell autonomous and paracrine signalling?

Although the studies described above fit with a genomic mechanism, two distinct non-genomic mechanisms have been reported that reveal both cell autonomous and paracrine signalling by glucocorticoids in the anterior pituitary (Figure 2). Importantly, the time to onset of these non-genomic inhibitory mechanisms overlaps with that observed for the genomic mechanisms above (Figure 1c).

### Rapidly reversible non-genomic CORT signalling in corticotrophs: Evidence for changes in CORT feedback sensitivity?

A rapid, reversible non-genomic mechanism of glucocorticoid inhibition has been identified in perifused cultured rat corticotrophs exposed to a constant low (30pM) exposure to CRH for > 1 h [49]. In cells that had been maintained in glucocorticoid free medium for > 6 h, exposure to 10 nM corticosterone 1 h after the start of the CRH infusion rapidly (∼5 min) inhibited CRH-stimulated ACTH secretion. This inhibition has several distinct features from the genomic mode of early glucocorticoid regulation of secretagogue evoked pulses of ACTH release discussed above. Firstly, inhibition was rapidly reversed within 5 min of washout suggesting constant exposure to CORT is required for inhibition and thus implying that sustained activation of signal transduction cascades is not key. Although inhibition was blocked by the GR antagonist RU38486, prior exposure to actinomycin D had no effect, supporting a non-genomic mechanism. In addition, a second ‘pulse’ of 10 nM glucocorticoid during the sustained exposure to CRH resulted in another transient inhibition of ACTH secretion. Application of 10 nM corticosterone to isolated rat corticotrophs, approximately 5 min after the start of a 100 pM CRH infusion, had no effect on intracellular free calcium levels up to 15 min of continuous CRH and CORT exposure, suggesting that, under these conditions, CORT acts distal to calcium entry.

However, this inhibitory mechanism was not observed in cells that had only been exposed to glucocorticoid-free medium for 2 h. Indeed, under these conditions only a 30 min application of corticosterone at 1uM, but not 10–100 nM, applied 1 h after the start of the constant CRH infusion, resulted in a slow inhibition that reached significance only after 30 min of corticosterone exposure [49]. Whether this lack of inhibition is a consequence of the long duration exposure to CRH, or reflects a dramatically altered sensitivity to CORT in these cultured corticotrophs, remains to be determined.

This non-genomic, rapidly reversible effect of corticosterone has been interpreted as a possible mechanism underlying ultradian control of ACTH secretion. Conceptually corticotrophs may be exposed to low (∼10 nM) CORT for several hours during the circadian nadir thus such a mechanism may explain initiation of ultradian rhythmicity at the start of the activity phase. However, loss of this rapid mechanism in cells that have recently (within 2 h) been exposed to higher levels of corticosterone would argue against a key involvement in controlling subsequent ultradian pulses later in the circadian peak. Importantly, these studies do provide important insight that sensitivity of corticotrophs to glucocorticoids may change depending on their prior exposure to glucocorticoid, in agreement with a wide body of evidence showing variation of stress responsiveness during the ultradian and circadian cycle [2,3].

### Non-genomic CORT regulation via folliculostellate cell paracrine signalling?

An additional non-genomic mechanism of early glucocorticoid inhibition has also been proposed through glucocorticoid induced release of the calcium and phospholipid binding protein annexin 1 (ANXA1) from folliculostellate (FS) cells [50,51]. FS cells form a complex interdigitating network in the anterior pituitary [52]. *In vitro* and *in vivo* studies in rats, utilising both immunoneutralisation and exposure of isolated pituitary tissue in static culture assays to ANXA1 and N-terminal peptides, implicated ANXA1 as a glucocorticoid-regulated paracrine inhibitor of anterior pituitary cell secretion of ACTH, PRL and TSH [39,53,54]. ANXA1 inhibits ACTH, PRL and TSH secretion evoked by either supraphysiological concentrations of CRH and VIP respectively, or forskolin, that stimulate the cAMP-pathway. Intriguingly ANXA1 did not inhibit TRH-induced PRL release, and while the effects of ANXA1 on AVP-stimulated ACTH secretion are unknown, this may suggest that ANXA1 does not mediate paracrine regulation of secretion stimulated by the PLC/PKC pathway. However, as TRH-induced secretion of TSH was inhibited by ANXA1 in this system the effect of ANXA1 may be cell type specific.

Glucocorticoid regulation of ANXA1 in FS cells appears to be biphasic. In both acutely isolated rodent pituitary segments and the immortalised TtT/GF FS cell line, glucocorticoids (within tens of minutes) induce serine phosphorylation and externalisation of ANXA1 from FS cells, via a largely transcription/translation independent mechanism [55]. ANXA1 is externalised by a non-conventional export pathway that is inhibited by glyburide, supporting a role for an ATP-binding cassette transporter, although mice globally deficient for ABCA1 are not defective for ANXA1 release [56]. Pharmacological inhibition studies suggest a role for protein kinase C, PI3 kinase and MAP kinase in glucocorticoid-induced externalisation, although whether these pathways are required for non-genomic glucocorticoid signalling *per se*, or control steps in ANXA1 externalisation, is not known [55,57]. For example, while rapid (within 15 min) activation of the ERK/MAPK pathway by glucocorticoids has been reported in the rodent hippocampus activation is only observed after 3 h following glucocorticoid exposure in anterior pituitary AtT20 cells [58,59]. Over ∼1–2hr, glucocorticoids increase transcription/translation to replenish intracellular ANXA1 stores in FS cells. ANXA1 has been proposed to mediate its inhibitory effects on corticotrophs through activation of the formyl-peptide family of G-protein coupled receptors (FPR), although pharmacological and siRNA experiments suggest this is not mediated by Fpr1 in rodents [50]. However, although the mechanism whereby signalling downstream of the FPR-family GPCRs results in inhibition of cAMP- evoked secretion is unknown, regulation of actin cytoskeletal dynamics has been proposed to inhibit secretion evoked by supraphysiological levels of CRH.

In mice with a global deletion of ANXA1, circulating levels of ACTH and corticosterone are unaffected, although in male, but not female, mice there is a significant increase in pituitary ACTH content and an almost 4-fold increase in corticotroph number [60]. Deletion of ANXA1 had no effect on the number of FS or other pituitary cells, although FS cell hypertrophy was apparent in males and females, and a decrease in pituitary PRL content has been reported [50]. Although direct analysis of rapid/early glucocorticoid feedback inhibition has not been reported from ANXA1 knockout mice, these data suggest that feedback *per se* is not altered. Moreover, early glucocorticoid inhibition is evident in AtT20D16; 16 corticotrophs [20] that do not express ANXA1, as well as in isolated dispersed cells at low density where paracrine/juxtacrine signalling is unlikely (e.g. the studies by Duncan et al., Romanò et al. [26,35]). This suggests that glucocorticoid mediated ANXA1-paracrine signalling in the pituitary is redundant for normal homeostatic regulation of ACTH release. Glucocorticoid regulation of the ANXA1 pathway may thus be more important for controlling anterior pituitary cell function in other contexts, for example, where suppression across multiple cell types [61] or at the pituitary network level is required [62]. FS cells also express a number of other paracrine signalling molecules. However, whether such paracrine signalling is important, as implicated in fast glucocorticoid and cannabinoid signalling in hypothalamic neurones [63], remains an open question [52].

### Mechanism(s) of non-genomic regulation

For both non-genomic mechanisms discussed above, the effects of CORT are blocked by GR antagonists and it has been proposed that inhibition is distal to calcium influx. This suggests potential direct non-genomic effects on the secretory machinery and/or actin cytoskeletal reorganisation [49,50,64]. Although this remains to be established, the diverse modes of non-genomic glucocorticoid signalling proposed in other systems appear to be very cell context specific [45]. Moreover, some reports *in vivo* in rats suggest that non-genomic feedback at the pituitary is not blocked by GR antagonists and is mimicked by aldosterone [65]. Conceptually non-genomic signalling by glucocorticoids in the pituitary may involve either extracellular or intracellular receptors, but the nature and identity is unknown. Glucocorticoids at sub-micromolar concentrations may directly regulate ion channel activity in some anterior pituitary cells [66], but this would be largely counter to the distal effects on calcium influx observed. Recent work has also revealed that GPR97, a member of the human adhesion GPCR family, is activated by cortisol and dexamethasone although its functional relevance in the pituitary is not known. However, GPR97 is unlikely to play a role in rapid glucocorticoid feedback as it is not activated by corticosterone and is also activated by inactive glucocorticoids such as cortisone [67]. Non-genomic signalling has also been proposed via glucocorticoid binding to the intracellular GR complex itself. Some studies have suggested that a fraction of the GR population is plasma membrane associated in an AtT20 variant (most likely D1 strain that grows in suspension but cells selected for cell adherence) [49]. The mechanisms by which GR associates with the plasma membrane remain to be resolved. For example, in the LβT2 gonadotroph cell line and HEK293 cells, acyl–biotin exchange reveals that the GR can be post-translationally modified by S-acylation, as for the estrogen receptor [68]. Cysteine residues that are S-acylated in GR have not been identified. Mutation of the consensus cysteine residue, that is, also conserved in estrogen receptors, to alnine reveals this cysteine residue is not the site of S-acylation. Moreover, the alanine mutation has no effect on GR membrane association [68]. In contrast, metabolic labelling studies failed to identify S-acylation of GR in the rat hypothalamic cell line 4 B [69], that displays membrane associated GR. In a number of systems, GR has been reported to be associated with membrane caveolae [45] revealing a range of potential mechanisms to control membrane association of GR depending on cell context. The cytosolic GR exists in a complex with a range of chaperones and other proteins, including protein phosphatase 5, with ligand binding changing protein composition upon nuclear translocation of the complex [70]. In the pituitary, although some non-genomic signalling cascades activated downstream of GR have been reported, such as src, ERK and CamKinase, they do not appear to be important for rapid non-genomic glucocorticoid inhibition of ACTH secretion *per se* [49].

Intriguingly, recent work in immortalised ND42 hypothalamic and isolated rat hypothalamic neurones suggests interplay between membrane glucocorticoid receptor signalling and conventional GR-induced transcription via the intracellular GR [71]. Tasker et al. revealed that a plasma membrane receptor for GR with an extracellular binding site can also rapidly induce (within 5 min) translocation of intracellular GR to the nucleus (presumably in the unliganded state) [71]. Importantly, these studies used denatured DEX-BSA to remove non covalently bound dexamethasone trapped in folded BSA to ensure DEX could only be presented extracellular by excluding any free DEX from the BSA conjugate – a major criticism of using such conjugates to assign membrane signalling in the past. This cell surface signalling by DEX-BSA was not blocked by a range of protein kinase inhibitors, but was blocked by an activator of Akt. Intriguingly, DEX-BSA resulted in GR-mediated transcriptional changes that were distinct from those regulated by DEX induced ligand activated GR, including that the DEX-BSA pathway did not activate a synthetic GRE reporter. If such a mechanism also exists in pituitary cells, this would suggest a mechanism by which glucocorticoids could exert distinct transcriptional responses depending on whether the cell-surface (extracellular) or classical intracellular glucocorticoid receptor is activated. Importantly, this might also reveal a continuum between non-genomic and genomic mechanisms of rapid and early glucocorticoid inhibition.

## Long-term corticosterone effects on anterior pituitary function

Glucocorticoids exert long term effects on anterior pituitary function on the timescale of hours to weeks, as a result of chronic stress or long-term exogenous glucocorticoid therapy. However, mechanisms underlying direct long term effects of glucocorticoids at the pituitary level are still relatively poorly understood. Indeed, effects of glucocorticoids on pituitary corticotrophs may be indirect including through regulation of hypothalamic input as well as changes in sensitivity of glucocorticoid feedback. Long term effects are generally ascribed to the well characterised genomic regulation of POMC expression and biosynthesis, as well as changes in GPCR expression [2,3,6,72]. Importantly, chronic stress can result in enhanced corticotroph output followed by a period of refractoriness that can take weeks for recovery [73]. Recent mathematical modelling supports a model in which changes in GR feedback, including at the pituitary, can result in temporal changes in functional pituitary gland mass – reflecting either changes in the number of corticotrophs or in the efficiency of stimulus secretion coupling [73]. In rats exposed to a chronic variable stress model, basal and CRH-induced ACTH release is enhanced *in vitro* and *in vivo,* revealing a sensitisation of corticotrophs at the end of the chronic stress [74]. Using a 2-week chronic stress model in Pomc-GFP mice, corticotroph spontaneous and CRH-induced excitability is significantly elevated in part dependent on enhanced pseudo-plateau bursting. This bursting is due to functional BK channels [75], supporting the hypothesis that chronic stress results in intrinsic changes in membrane excitability. Whether this enhanced excitability is a direct result of glucocorticoid signalling at the pituitary corticotroph *per se*, or whether corticotroph excitability is suppressed during recovery from the chronic stress, is not known. Recent single-cell RNA-seq in mice, and proteomic analysis in rats exposed to chronic stress paradigms reveal relatively small changes in gene and protein expression associated with a variety of cellular signalling pathways [76,77]. Intriguingly, a 4-week dexamethasone treatment to pomc-GFP mice resultant in significant changes in mRNA expression of a cohort of ion channels and transporters, as well as components of cognate signalling pathways, specifically in corticotrophs [78]. This suggests that changes in cell signalling and membrane excitability may be a common mechanism that contributes to changes in corticotroph function during chronic stress, long-term glucocorticoid treatment, as well as following recovery.

Taken together this suggests that control of anterior pituitary corticotroph excitability may be a common target for both short- and long-term regulation of corticotroph physiology. Importantly, as most pituitary cells are electrically excitable the role of glucocorticoid regulation of excitability in other cell types warrants further investigation and promises a potential approach to both diagnose and treat pituitary related disorders.

## Conclusions & perspectives

Anterior pituitary cells have to be able to respond appropriately to dynamic pulses of glucocorticoid that vary in timing, amplitude and duration under non-stressful and stressful conditions. This adaptability provides a clear rationale as to why glucocorticoids utilise different mechanisms for temporal control of anterior pituitary function across a wide dynamic range. Understanding the context in which these signalling cascades operate is essential for our understanding of glucocorticoid and anterior pituitary physiology, and many open questions remain.

An emerging feature is that a key overarching mechanism for regulation in both early and late phases of glucocorticoid regulation is through control of membrane excitability and calcium signalling, that may be of relevance across multiple excitable pituitary cell types. While specific ion channels may be targets in some contexts (e.g. role of BK channel in CRH-dependent bursting), evidence supports that glucocorticoids can control excitability and calcium signalling through multiple mechanisms and cellular targets. In part, this likely reflects that different signalling pathways can be engaged to stimulate secretion (e.g. differences in regulation of CRH vs. AVP), depending on physiological context. A major challenge is to define glucocorticoid proteins and their targets that underly early glucocorticoid inhibition, as well as understanding the role of direct vs. indirect effects of longer term changes in glucocorticoid, such as in chronic stress or glucocorticoid treatment. Intriguingly, glucocorticoids also control intrinsic excitability of CRH neurones that can be antagonised by potassium channel inhibitors [9], suggesting control of excitability is a common general mechanism at multiple levels of the HPA axis.

Elucidation of the molecular nature of membrane receptors that mediate rapid non-genomic effects of glucocorticoids in pituitary cells is required: does this include a role for post-translationally modified classical GR or distinct GPCR receptors? The intracellular signalling cascades activated by membrane GR signalling to rapidly inhibit secretion and the importance of paracrine signalling in non-genomic glucocorticoid action remain to be determined. Moreover, the potential for a continuum and crosstalk of non-genomic and genomic mechanisms to regulate anterior pituitary function, from minutes to weeks, remains to be explored, including the potential role glucocorticoid mediated regulation of miRNA expression in the anterior pituitary [79]. In addition, as the anterior pituitary receives regulatory input from the brain and periphery, understanding how glucocorticoids regulate pituitary cell function via effects on non-pituitary sites is key, including the identity of genes regulated directly or indirectly by glucocorticoids.

The mechanistic studies described here are largely derived from analysis of pituitary cells *in vitro* and a significant challenge for the future is understanding the importance of these mechanisms *in vivo.* For example, we know almost nothing about how corticotroph electrical excitability and calcium signalling are regulated *in vivo*, by secretagogues or glucocorticoids. Furthermore, as corticotrophs, as other pituitary cells, form anatomical networks *in vivo,* it remains to be determined whether glucocorticoids control function at the network level, perhaps through FS cells. In addition, the dynamics of functional glucocorticoid action at pituitary cells *in vivo*, as well as how pulses of hormone are coordinated across pituitary populations, is poorly understood. In this regard, multiple factors are likely to control the dynamics of exposure to glucocorticoids, as well as the functional effect of glucocorticoids on secretion from any particular pituitary cell population, including: i) the control of pituitary blood flow and anterior pituitary cell location relative to capillaries [62] ii) the presence of glucocorticoid binding proteins and metabolising enzymes controlling the level of biologically active glucocorticoid [6,80], iii) the expression of glucocorticoid receptors and cognate signalling pathways, iv) network behaviour and cross-talk including by paracrine signalling between pituitary cells.

Defining the dynamics and associated mechanisms of glucocorticoid action at the anterior pituitary is key for our understanding of the normal physiological function of glucocorticoids, how disruption to these dynamics contributes to human disease and how we can best diagnose and treat glucocorticoid and stress related disorders. We are still only at the foothills of our understanding – these are major questions, and opportunities, for the community to address in the future.

## Conflict of interest statement

Nothing declared.
